# 

**Published:** 1897-06-19

**Authors:** 


### EARLY EDITION.

**Notices of Births, Deaths, and Marriages** are inserted in this column at a charge of 2s. 6d. for an announcement not exceeding four lines.

**DEATH.**

On the 7th inst., Elizabeth Willis Skidmore, late "Sister" in the Royal Infirmary, Sheffield. (1,574)

### NOTICE TO CORRESPONDENTS.

All MS., Letters, Books for Review, and other matters intended for the Editor should be addressed The Editor, "The Hospital," The Hospital Building, 28 & 29, Southampton Street, Strand, W.C.

The Editor cannot undertake to return rejected MS., even when accompanied by stamped directed envelope.

**Notice to Advertisers and Subscribers.**

All Advertisements, Orders for Copies of The Hospital, and Business Communications should be addressed to **THE MANAGER, (Not to The Editor),** The Hospital, The Hospital Building, 28 & 29, Southampton Street, Strand, London, W.C.

The Cost of Subscription, post free, is as follows:—Per week, 2½d.; per quarter, 2s. 8d.; per half-year, 5s. 3d.; per annum, 10s. 6d. Foreign:—Per annum, 15s. 2d.; payable, in all cases, in advance.

**NOTICE.—Vol. XXI. of "The Hospital" (October, 1896, to March 1897), in handsome cloth binding, is on sale at the Office of this Journal, price 7s. 6d. Cases for binding the Numbers contained in this Volume, 1s. 6d. Index to Vol. XXI., 2½d., post free.**

**The Monthly Part for June is now ready. Price 6d., by post 8d.**

### BOOKS RECEIVED.

Isbister and Co.

"St. Paul's Cathedral." By the Rev. W. C. E. Newbolt. Illustrated by Herbert Railton.

"Ely Cathedral." By the Rev. W. E. Dickson. Illustrated by Alexander Ansted.

The Sanitary Publishing Co.

"Notes on Practical Sanitary Science." By William H. Maxwell.

The Cotton Press.

"Sanitary and Social Questions of the Day."

Cassell and Co.

"The Surgical Diseases of Children." By Edmund Owen, M.B., F.R.C.S.

Bailliere, Tindall, and Cox.

"Clinical Manual of Mental Diseases." By A. Campbell Clark, M.D.

**Periodicals and Pamphlets Received.** — Mothers and Daughters, The London Argus, The Queen's Empire, The Literary Digest, The Woman's Signal, Health News, "Queen of Queens," Girl's Own Paper, Medical Record, Sunday at Home, Sunday Hours, The Leisure Hour, Boy's Own Paper (Record Summer Number), Boy's Own Paper, Westminster Review.

### THE STUDENT OF MEDICINE.

**APPOINTMENTS.**

H. Brown, M.D.Lond., appointed Medical Officer to the Ipswich and East Suffolk Hospital. C. B. Cranstoun, M.B.Durh., appointed Medical Officer of Health for the Borough of Ludlow. H. D'Arcy Ellis, L.R.C.P.Edin., M.R.C.S.Eng., appointed Medical Officer of Health to the Kingswinford Rural District Council. Dr. W. Fry, appointed Medical Officer for the East Peckham District of the Malling Union. C. B. Gittings, M.B.Lond., appointed Resident Medical Officer to the St. Marylebone General Dispensary. J. G. Havelock, M.D.Edin., appointed Physician Superintendent to the Royal Asylum, Sunnyside, Montrose. E. S. Hawthorne, L.R.C.P.&L.M., L.R.C.S.& L.M.I., appointed Surgeon to the Etheridge District Hospital, Georgetown, Queensland. J. Henry, M.D.R.U.I., M.Ch., appointed Medical Officer for the Monoghan Dispensary District. Dr. Higgins, appointed Medical Officer for the Dungiven Dispensary District. C. T. W. Hirsch, M.R.C.S.Eng., L.R.C.P.Lond., L.S.A., appointed Divisional Surgeon to the police of R Division stationed at Woolwich, and to police of Woolwich Division stationed at the Royal Arsenal Railway Plant Depôt and Woolwich Dockyard. J. C. Howden, M.D., appointed Consulting Physician to the Royal Asylum, Sunnyside, Montrose. J. C. R. Husband, L.R.C.P.Edin., M.R.C.S.Eng., appointed Medical Officer for the Workhouse and to the No. 2 District of the Ripon Union. A. MacLennan, M.B., C.M.Glasg., L.M.RotundaHospitalDub., appointed Resident Medical Officer to the Hospital for Women, Soho Square, W. J. M'Donnell, L.R.C.P.I., L.R.C.S.-Edin., appointed Medical Officer for the Roscommon Dispensary District. P. T. O'Sullivan, M.B., B.Ch., R.U.I., appointed Physician to the South Charitable Infirmary and County Hospital, Cork. J. S. Pickford, L.R.C.P.Lond., M.R.C.S.Eng., appointed Medical Officer of Health to the Little Lever Urban District Council. J. S. Rankin, M.B., C.M.Glasg., appointed Medical Officer for the Central District of the Govan Parish Council. J. Robertson, M.D Edin., appointed Medical Officer of Health for Sheffield. W. A. Skinner, M.B.C.M.Edin., appointed Junior Assistant Medical Officer to the Royal Asylum, Sunnyside, Montrose. Dr. C. Stewart, appointed Medical Officer for the Parish of Kilmaurs. H. M. Sylvester, M.R.C.S., L.R.C.P.Lond., appointed Medical Officer of Health to the Leiston-cum-Sizewell Urban District Council. M. Thorne, L.S.A.Lond. M.B.Brux., appointed Senior Resident House Surgeon to the New Hospital for Women.

